# A generative adversarial network with “zero-shot” learning for positron image denoising

**DOI:** 10.1038/s41598-023-28094-1

**Published:** 2023-01-19

**Authors:** Mingwei Zhu, Min Zhao, Min Yao, Ruipeng Guo

**Affiliations:** College of Automation Engineering, Nanjing University of Aeronautics and Astronautics, CO, Nanjing, 211106 People’s Republic of China

**Keywords:** Engineering, Optics and photonics

## Abstract

Positron imaging technology has shown good practical value in industrial non-destructive testing, but the noise and artifacts generated during the imaging process of flow field images will directly affect the accuracy of industrial fault diagnosis. Therefore, how to obtain high-quality reconstructed images of the positron flow field is a challenging problem. In the existing image denoising methods, the denoising performance of positron images of industrial flow fields in special fields still needs to be strengthened. Considering the characteristics of few sample data and strong regularity of positron flow field image,in this work, we propose a new method for image denoising of positron flow field, which is based on a generative adversarial network with zero-shot learning. This method realizes image denoising under the condition of small sample data, and constrains image generation by constructing the extraction model of image internal features. The experimental results show that the proposed method can reduce the noise while retaining the key information of the image. It has also achieved good performance in the practical application of industrial flow field positron imaging.

## Introduction

Positron Emission Tomography (PET) can be used to detect the flow field in the cavity of complex industrial parts. The reconstructed flow field image can describe the internal state of the cavity and help experts to judge and eliminate faults. In practice, due to the inherent imaging characteristics of the technology, the existence of noise and artifacts is inevitable. The main reasons are as follows: (1) The limitation of imaging system hardware equipment: this type of noise cannot be avoided and eliminated, and the process is not subject to human intervention; (2) There are a large number of response lines in the original PET sampling data with zero counts: this type of response line is in the Speckle background noise will be generated during the reconstruction process; (3) The reconstruction process includes the influence of other factors such as algorithm and parameter selection.

The noise in positron flow field image is unnecessary or redundant interference information, which will directly interfere with the judgment of industrial failures. Therefore, to obtain a clean positron flow field image, it is necessary to denoise the original image. technology.

In recent years, researchers have devoted themselves to studying the application of positron imaging technology in industrial non-destructive testing. Maximum Likelihood Expectation Maximization (MLEM) is the current general algorithm for positron image reconstruction^1^. The optimization of loss function^2^, improvement of statistical model^3^ and introduction of prior knowledge^4^ of the algorithm have improved the quality of reconstructed images to varying degrees, but there are still loss of data details and artifacts.In addition, the existing iterative reconstruction algorithms have relatively high requirements for computing costs, which do not meet the actual industrial application scenarios.

On the other hand, to improve the image quality, many researches directly pre-filter the sinusoidal data in the original sampling, and model the sinusoidal data to obtain the noise characteristics for filtering^5,6^.

Therefore, in the image post-processing stage, it has higher research significance and practical application value to improve the image quality by denoising or artifact suppression of the reconstructed flow field image^7^. Realized the adaptive estimation of the image noise relationship by using the non-local mean algorithm to study the image redundancy information and optimize the non-local weights in the image^8^; used the knowledge of sparse learning, a method based on batch dictionary learning is proposed to suppress speckle noise and fringe artifacts in the reconstructed image^9^; realized the fast 3D matched filter, and removed the random noise to obtain a better signal-to-noise ratio effect in the reconstructed image.

Although some progress has been made in related researches, there is still a lack of targeted research on industrial reconstruction images. In practical applications, the sampling data of flow field positron images is low, and the requirement for texture details is high, which makes the existing denoising methods unable to meet the processing of such industrial positron images.

Therefore, to address the above issues, we propose a denoising model of a generative adversarial network incorporating zero-shot learning knowledge for denoising reconstructed images of positron flow fields in closed cavities for higher quality Image. Specifically, the contributions of this paper are as follows:

1. To our best knowledge, this is the first domain generative network model for image denoising of industrial positron flow field.
2. It realizes the image denoising under the condition of scarce data by adding feature input learning from the pixel information inside the images.
3. It constructs a new loss function, which combines perceptual loss and edge loss to preserve image details as much as possible.
4. It provides SOTA denoising results in the positron images in industrial flow field.

## Related work

### Image denoising based on neural network

Deep convolutional neural network (CNN) is the most popular network in the current image processing task. With the proposal of the network^10,11^, the overall implementation of CNN tended to be mature in the depth and width, so it shows a good effect in the image denoising task^12^. Trained a set of fast and effective convolutional neural network fusion modules based on prior knowledge, which is not only effective in Gaussian noise but also suitable for low-level vision applications^13^. Used deep convolution networks to optimize the network and learn the end-to-end image mapping, to improve the image quality^14^. Proposed the denoising convolutional neural networks (DnCNNs), which used residual learning and batch normalized training networks for blind denoising, and realized the processing of Gaussian noise of different levels using a single network model.

### Generative adversarial networks

Generative adversarial network^15^ consists of two parts, the generative model *G* and the discriminative model *D*. The input random noise generates realistic images through the generative network training, at the same time, the discriminative network distinguishes the true from the false. The mathematical model is shown in Eq. (1).

1 $$\begin{aligned} \begin{array}{r} \min _{G} \max _{D} V(G, D)=\min _{G} \max _{D} E_{x \sim P_{\text{ data } }}\left[\log D(x)\right] +E_{z \sim P_{z}}\left[\log (1-D(G(Z)))\right] \end{array} \end{aligned}$$

Here, $\mathbb {E}(\bullet )$ represents the expectation operator; *x* represents the real data and *z* represents the input random noise. When *D* is trained as the optimal discriminator, which means the *JS* divergence is minimized, and the training for *G* is completed, the optimal data generation network can be obtained.

Generative adversarial model can supplement training data and have achieved good performance in few-sample tasks. The proposal of papers^16,17^ further provided possibility for the specific implementation of the model, including the researches on network convergence, model collapse, and the optimization of the loss function. These researches all achieved good performances in many fields such as image super-resolution^18^, image transformation^19^, image style transfer^20^. The combination of the model and convolutional neural network also shows excellent performance in the task of image denoising^21^. Trained the two networks jointly, and the voxel loss function is constructed to realize image denoising and obtain a high peak signal-to-noise ratio^22^ highly under-sampled data to reduce the artifacts and contrast, which improved the image quality under the framework of the conditional generative network^23^. Used GAN to model the noise distribution to generate noise samples, and formed a clean image set as training data. The network had trained for blind denoising and achieved well results^24^. Rendered small pixel samples using the features of GAN and obtained the higher quality real images by training the noisy images.

### “Zero-shot” learning

The limitation of deep neural network is that it needs enough sample data to train a good network model. Therefore, when dealing with small sample data, to obtain a good model through training, we consider learning the attributes of existing samples, and then using the knowledge of partial transfer learning to identify the type attributes of unknown data. “Zero-shot” learning^25^ is an unsupervised learning network based on zero samples, and the original core idea is to realize the transfer learning of unknown data by learning the attributes and labels of existing samples. In recent years, great progress has been made in the research of related networks^26^. Improved the original model and trained a labeling framework for model embedding directly, which realized the prediction of data categories^27^. Solved the problem of domain drift by adding new constraints in the network and it can ensure the original visual feature information while semantic embedding.

At the same time, due to the excellent performance of “zero-shot” learning in the unsupervised field, more and more researches on models that integrate “zero-shot” learning under the framework of generative adversarial network are also gradually carried out^28^. Proposed the loss function of gradient signal to solve the problem of zero-shot learning by generating data samples simulating unsupervised learning in a generative adversarial network^29^. Used the Coupled GANs extension as conditional GANs, which can capture the joint distribution of domain adaptation samples in different tasks, and complete the adaptive domain training. The above papers show that it is feasible to embed a zero-shot learning module in GAN framework, and some achievements have been achieved.

Therefore, we consider denoising the industrial positron flow field image by integrating the knowledge of “zero-shot” learning in the framework of generative adversarial network. The rest of this paper is organized as follows. The proposed method is introduced in Section "Method". The experiments and results are presented in “Experiments" Section. Finally, relevant issues are discussed and conclusions are drawn in “Conclusions" Section.

## Method

### Image feature learning

The data set of natural images is easy to obtain, and the performance of the neural network model can be improved through large-scale data training. However, the positron emission tomography image of industrial flow field belongs to the research object of scarce sample data. The research on the application of positron emission tomography imaging technology in the field of industrial flow field detection is still in the preliminary stage, with strong data field characteristics and high sampling difficulty, resulting in less sample data and difficult to obtain. Therefore, in the absence of a sufficient number of training samples, we plan to divide the image through the repeatability characteristics of the internal pixels of a single image, and extract the internal features of the image in a small enough scale. In the actual industrial application scenario, the positron flow field image is greatly affected by the environment generated by the flow field. Even for the same industrial part, different reconstruction images will be obtained due to different usage scenarios. In this case, a considerable part of the image information is not easy to obtain from the external image data. At the same time, considering the regularity and repeatability of the industrial flow field image itself, it is necessary to obtain more image features by learning the internal information of the image, so as to avoid the loss of details in the denoising process. The experiments in the paper^30^ show that the information entropy of the image is smaller than that of the image from the external data set. Furthermore, by observing the internal statistical information of the image, more accurate prediction results can be obtained compared with the external statistical information of the image.

**Figure 1:**
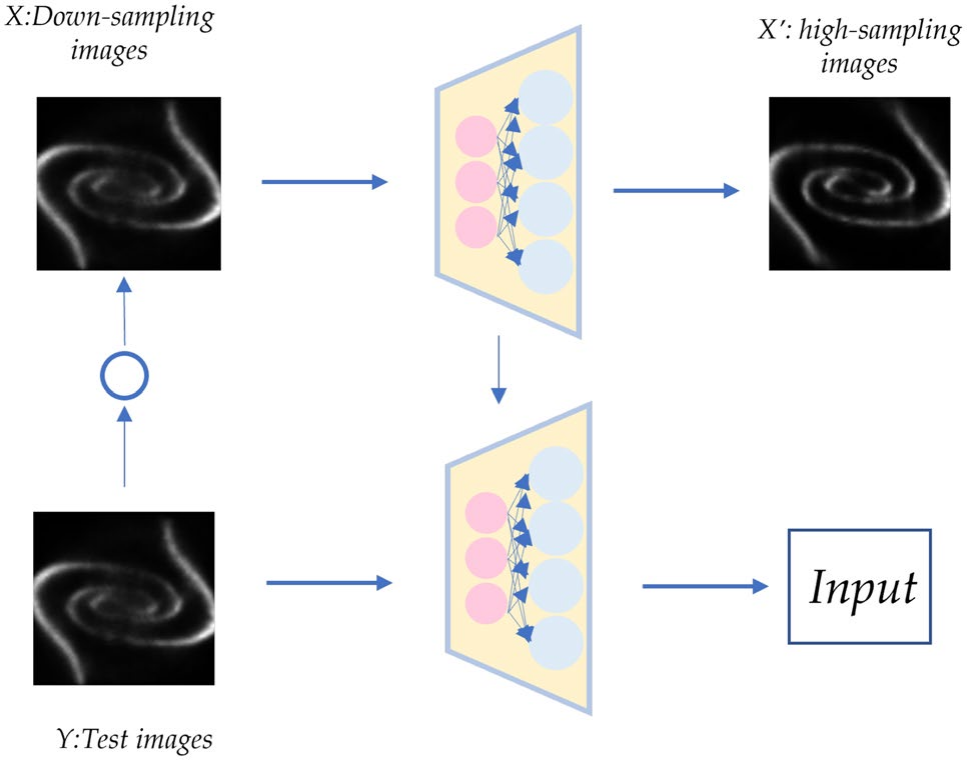
*X* represents the noisy image part of lower sampled data, $X^{\prime }$ represents a clearer part of the image; *Y* represents the test images. The proposed network model can learn a corresponding mapping relationship through the information extraction of pixels in the image, and applied to the test images on the right to obtain the output of clearer images.

Considering the above factors, we establish a feature extraction model of internal image information. The principle is based on “zero-shot” learning, and the purpose is to use a single small number of images for feature extraction in the case of small sample data. A prerequisite for the feasibility of this model is that the flow field image is different from the general image, and its own regularity is strong.The specific model construction process is as follows:first, a convolutional neural network needs to be trained, and small-scale image samples extracted from the flow field image are used as training samples. The image examples here are obtained by randomly slicing the flow field image. Then, by learning the mapping relationship between the area with high image noise and the position with low noise, a convolution network for learning the internal information of the flow field image is obtained. The network adopts a full convolution layer network structure, and each layer of the network is activated using the RELU (Rectified Linear Units) function. The corresponding relationship is shown in Fig. 1, and the aim is to obtain the feature space correspondence of the same category of images: $f_{z s l}: X \rightarrow X^{\prime }$. Here, since the network is trained by a single image, it can greatly reduce the training time and complexity of the network, realize the internal feature extraction of the image, and its output is used as a conditional input of the generative countermeasure network to construct the denoising model of specific images.

### Generative adversarial networks

**Figure 2:**
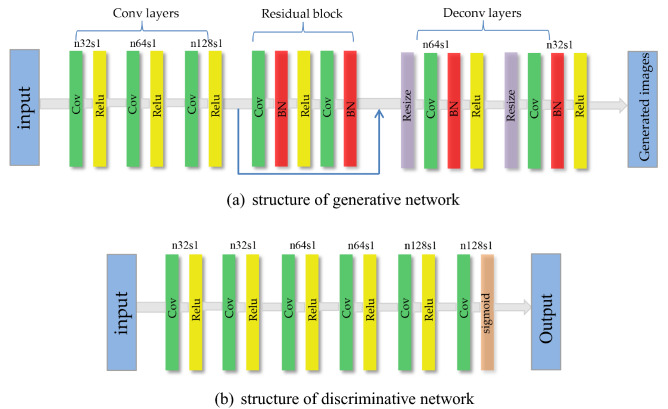
*n* and *s* mean the kernels and stride of the convolutional layer.

After extracting the internal features of the flow field image, a positron flow field image denoising model is built with the generative adversarial network as the model framework. The input of the network is the feature vector and random noise extracted in the convolutional network in the previous section and the overall network structure is constructed by the residual network (ResNet)^31^. The specific implementation is shown in Fig. 2: the generator is consist of convolution layers, residual blocks and deconvolutional layers. The kernel is $3 \times 3$, the output is a separate $3 \times 3$ characteristic graph, the stride is 1, the padding is 1 and the activation function is Rectified Linear Units (Relu). The discriminator is consist of six convolutional layers, which adopts the full convolutional networks, the kernel is $3 \times 3$, the activation function of the first five layers of convolutional network is Relu, and the last output layer is the sigmoid function.

### Loss function

Through the above steps, the positron flow field images with high noise are converted into clearer images. In addition to the basic adversarial loss function in the network, to obtain a better positron flow images denoising model, we consider constructing a new loss function to measure the performance of the denoising model. Firstly, to preserve the image information and detail features as much as possible in the denoising process, we add a perception loss function as shown as Eq. (2).

2 $$\begin{aligned} L_{\text{ pre } }=\mathbb {E}_{(x, z)}\left[ \frac{1}{w d h}\Vert G(z)-x\Vert _{F}^{2}\right] \end{aligned}$$

Here $\Vert \bullet \Vert$ represents Frobenius norm, *w*, *d*, *h* respectively represent the width, height, and depth of the feature space.

Additionally, to avoid excessive smoothing of the edge of the denoised image as much as possible, we give an edge loss function, and the mathematical expression of the function is shown as Eq. (3). Here, $\hat{x}$ represents the original images containing pixel feature information.

3 $$\begin{aligned} L_{{\text {mar}}}=\frac{1}{2} \sum \left(\widehat{x}-G(z)\right)^{2} \end{aligned}$$

Therefore, combined with the above two loss functions and the original adversarial loss function of the generative adversarial network, the overall joint loss function constructed in this paper is shown as Eq. (4).

4 $$\begin{aligned} L=\min _{G} \max _{D} v_{1}(D, G)+\lambda _{1} L_{p r e}+\lambda _{2} L_{{\text {mar}}} \end{aligned}$$

Here, $\lambda _{1}$ and $\lambda _{2}$ are weighted parameters, which weigh the weight between the three loss functions and the specific value is determined by the training effect of the network model in the actual process.

### Network framework

**Figure 3:**
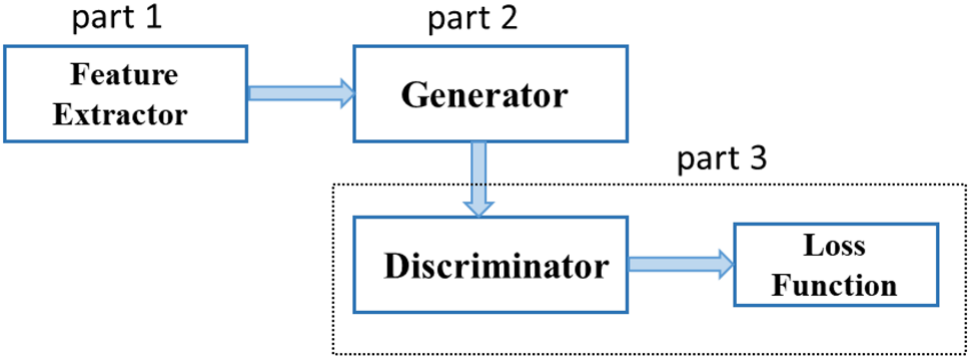
Network framework diagram of denoising model.

The generative adversarial model with zero-shot learning proposed in this paper is focused on positron flow field images in the industrial field and the overall framework is shown in Fig. 3. The model consists of three parts. The first part is the image feature learning network proposed in "Image feature learning" Section, which is composed of fully convolutional neural network. The second part is the generative adversarial network constructed in "Generative Adversarial Networks" Section. Here, the input of the generative network is the mapping relationship between the higher quality image and the noise image obtained in Fig. 1, which is the prior constraint. Then, we train the lower sampled noise image by the discriminative network and use the loss function defined above to characterize the model. Finally, we can obtain a denoising model on neural network for positron flow field images.

## Experiments

### Experimental data

The image data we used in the experiment is the positron flow field image in the industrial field. The data is obtained through GATE (Geant4 Application for Tomographic Emission) simulation. GATE is a special PET simulation software based on Monte Carlo. The specific simulation process is as follows: construct the geometric model of the detector; construct the geometric model of the scanned object; set particle transmission parameters; set the front-end electronic characteristics; set the data output format and obtain the data, and then the positron images are reconstructed by the algorithm. Here the reconstruction algorithm we used is MLEM.

We obtained twenty kinds of flow field images under different scenes, which contain different water medium equipment as much as possible, including water tunnels, tanks, and pipe flow devices of different specifications. At the same time, in the simulation process, we set two different standards of reagent dose and sampling time. In principle, the longer the sampling time, the higher the activity, and the better the quality of the reconstructed images.

### Network training

**Figure 4:**
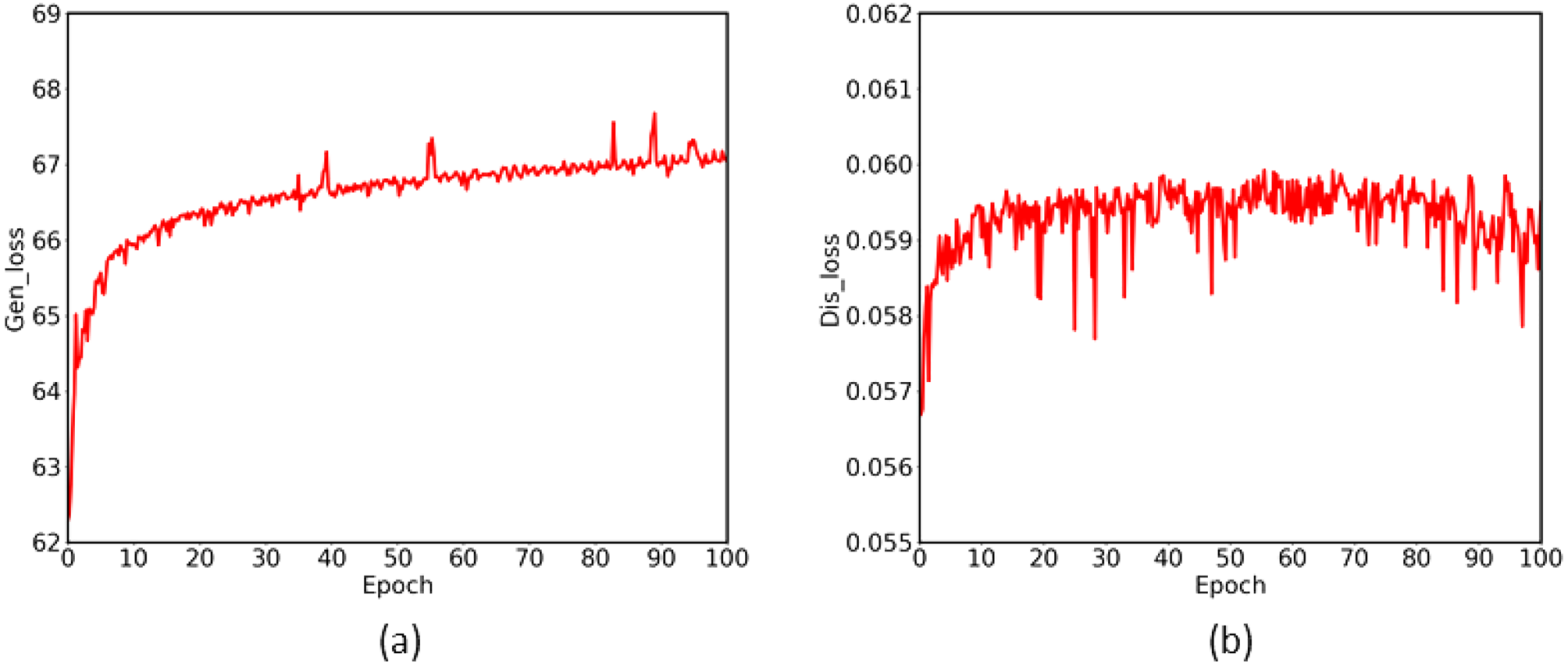
100 epochs are set in the training: (**a**) the loss function of the generative model; (**b**) the loss function of discriminative model. the whole model tends to converge around 80 epochs.

In our experiments, the model is shown in “Method” Section. All the networks were optimized using Adam algorithm^32^, and the hyper-parameters for Adam were set as $\alpha =1 \textrm{e}-5, \,\beta _{1}=0.2,\, \beta _{2}=0.9$. The networks were implemented in Python with Tensorflow, and the GPU used in the training is NVIDIA 2080Ti. We set the mini-batch is 64 in the process of the training. The loss function constructed above is shown in Fig. 4, and we can see the convergence of the model in the process of the denoising network for positron flow field images clearly.

**Figure 5:**
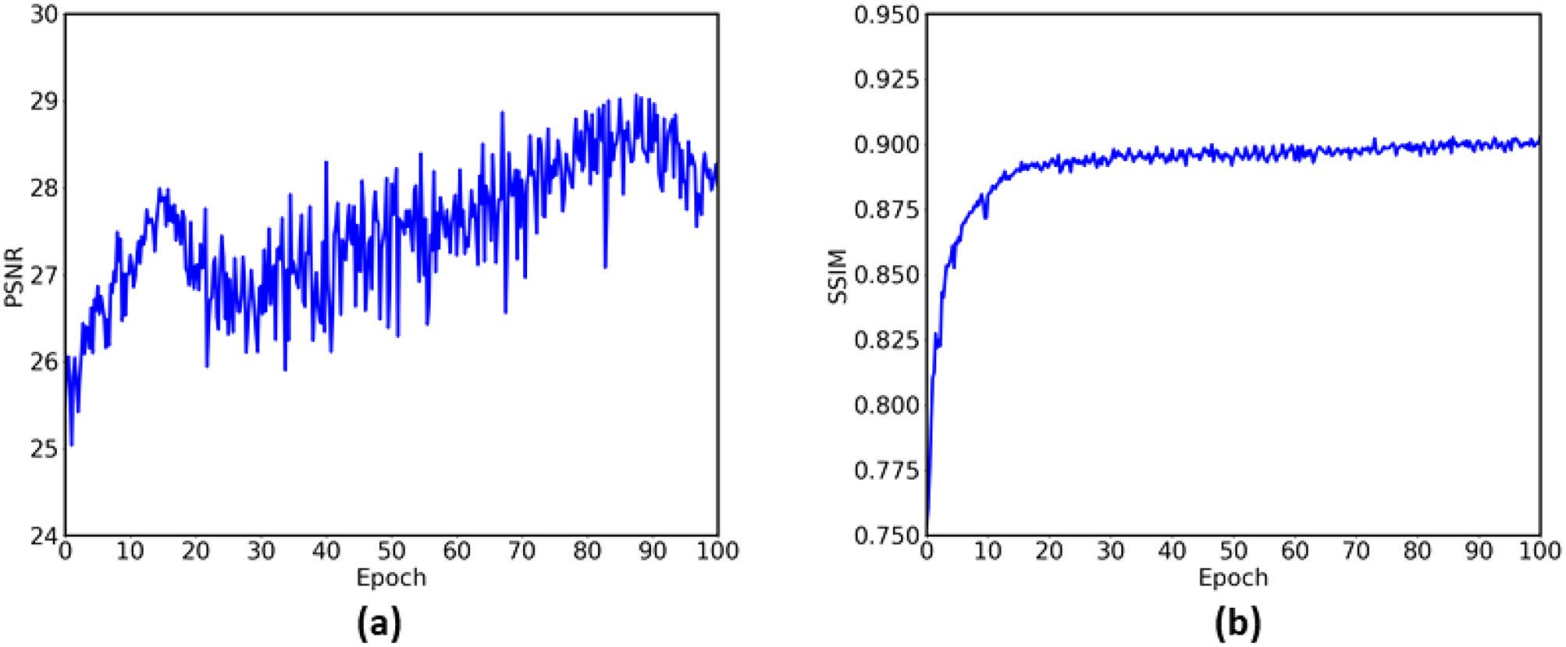
The two line chart of PSNR and SSIM are changed with the training. The reference image is a randomly selected image in the data set, which can basically reflect the changes of the image in the training process.

At the same time, to show the effect of the denoising model, the two quantitative indicators are used to measure the performance of the model synchronously, namely peak signal to noise ratio (PSNR) and structure similarity image measure (SSIM). These two are currently more common indicators and the mathematical expression is shown in Eq. (5), and the changes of values during model training are shown in Fig. 5.

5 $$\begin{aligned} \begin{aligned} M S E=\frac{1}{H W} \sum _{i=0}^{H} \sum _{j=0}^{W}\left\| X(i, j)-Y(i, j)^{2}\right\| \\ P S N R=10 \cdot \log _{10}\left( \frac{M A X_{I}^{2}}{M S E}\right) \\ S S I M=\frac{\left( 2 u_{X} u_{Y}+C_{1}\right) \left( 2 \sigma _{X Y}+C_{2}\right) }{\left( u_{X}^{2}+u_{Y}^{2}+C_{1}\right) \left( \sigma _{X}^{2}+\sigma _{Y}^{2}+C_{2}\right) } \end{aligned} \end{aligned}$$

### Experimental results

To show the denoising effect of the model on the positron flow field images proposed in the paper, we selected two different flow field slice images: laminar flow and turbulent flow. The model simulates two different fluid states due to the change of velocity in the same space. When the velocity is very small, the fluid flows in layers and does not mix; When the velocity increases, vortices will be produced in the flow field. Compare the effects of two flow field images under different denoising models. The basic description of the models used as the comparison is shown in Table  1. The first three models are the current general denoising models, and the last one is the ablation experimental model without changing the loss function.

**Table 1:** Description of comparative network model.

Model	Structure description
DnCNN^14^	CNN with MSE loss only
GAN^24^	GAN
CNN-GAN^19,23^	CNN with GAN
Zero-GAN	GAN + Zero-learning with original loss

**Figure 6:**
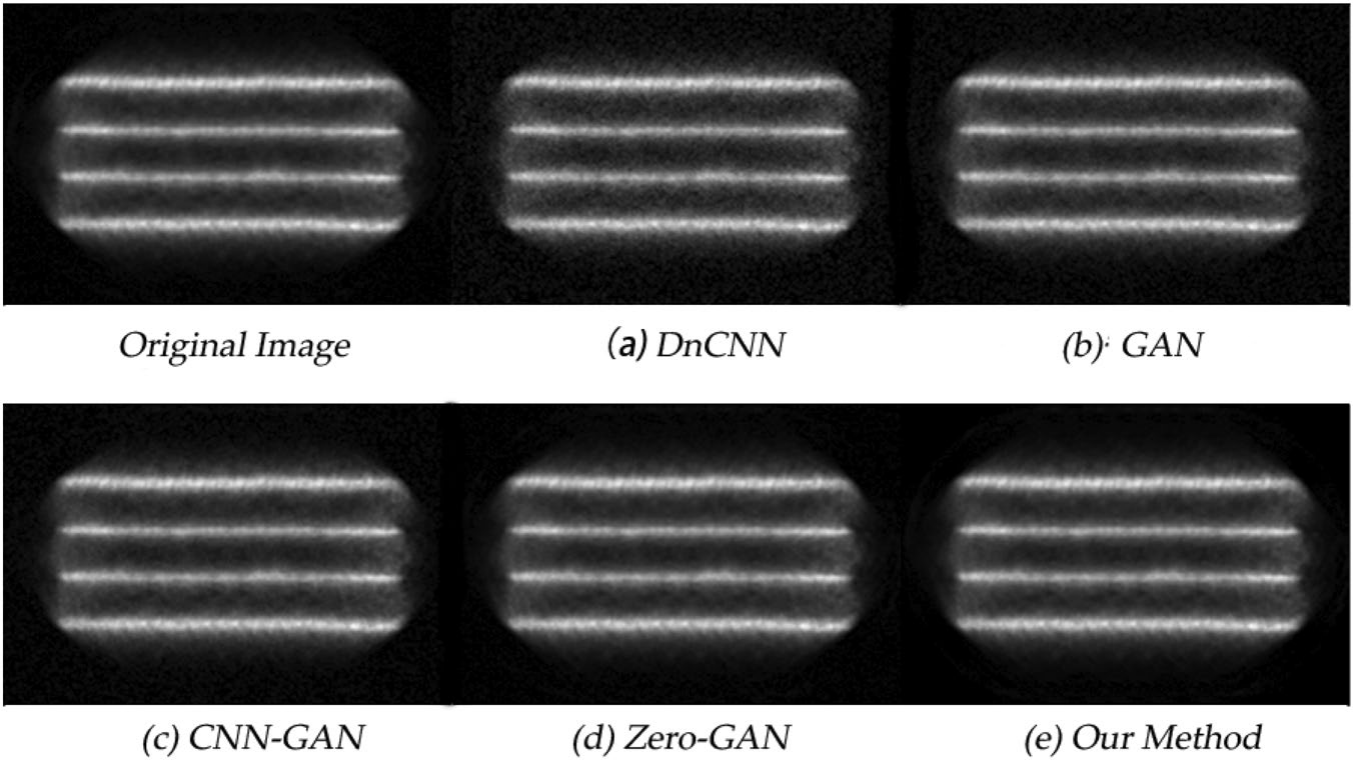
The positron flow field images are the laminar flow images obtained by simulation. The specific parameters are sampling time 1s and nuclide activity 800 bq.

**Figure 7:**
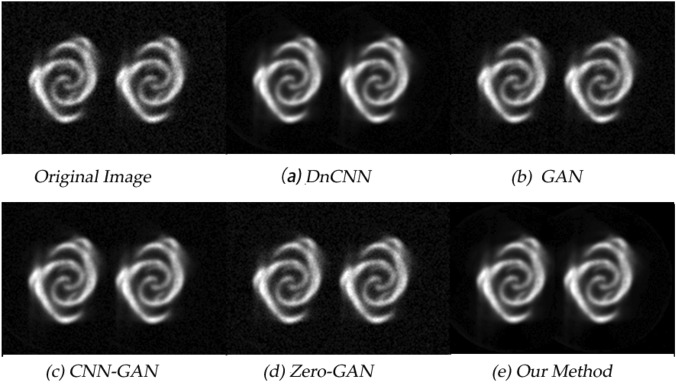
The positron flow field images are the vortex images obtained by simulation. The specific parameters are sampling time 1s and nuclide activity 800 bq.

The denoising results obtained under different network models are shown in Fig. 6 and Fig. 7 respectively. It is not difficult to find that all methods have a certain denoising effect on the positron flow field images. Compared with the original reconstructed images, the quality of the obtained images is improved to varying degrees, but obviously, the quality of the images obtained by the method proposed in this paper is the best after denoising.

### Quantitative analysis

Positron flow field images are the gray images obtained from the reconstructed sampling data. So the quality of the image cannot be accurately evaluated by human eyes alone, and there may be visual bias, especially in the details of the image. For quantitative analysis, we calculated the PSNR and SSIM, and the summary data are in Table  2.

**Table 2:** Quantitative results associated with different network outputs for Figs. 6 and 7.

	Fig. 6		Fig.7	
	PSNR	SSIM	PSNR	SSIM
DnCNN	27.872	0.804	27.954	0.704
GAN	28.326	0.635	27.898	0.683
CNN-GAN	27.928	0.856	30.127	0.742
Zero-GAN	29.061	0.892	29.568	0.837
Our method	29.342	0.897	31.783	0.925

From the values of the two indicators given in the table, it can be seen that the methods proposed in this paper have good performance. However, only using the generative countermeasure network to reduce the image noise is likely to generate beautiful positron images that do not conform to the characteristics of the industrial field, which cannot achieve the practical application effect. Therefore, the results further prove the necessity of constructing a new loss function.

At the same time, due to the objective conditions of the existing industrial positron imaging technology, image blur (blocky or smooth artifacts) may occur, which will also affect the results of quantitative evaluation indicators. Therefore, different from natural images, the application of positron flow field images in industrial non-destructive testing needs more expert experience to judge.

### Experimental verification

**Figure 8:**
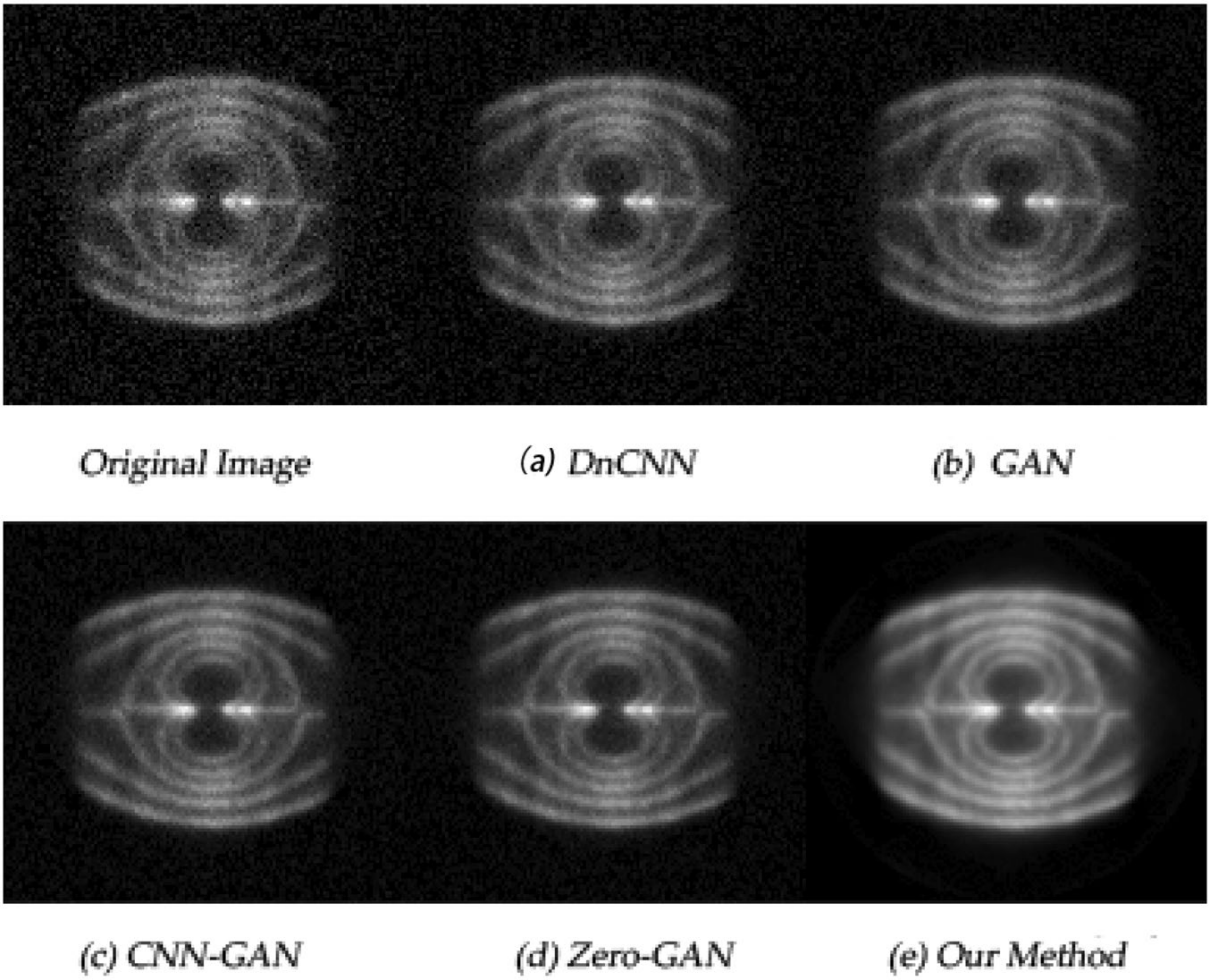
Fluid diagram of intertnal fluid in engine pipeline.

To further verify the advanced nature of the model, we designed the experiment as follows: it simulated the flow state of liquid in the engine pipe. The annular detector used is 64 detector rings with a radius of about 64 mm, and each detector ring is composed of 23 pairs of detector heads; the carrier solvent is water-soluble hydraulic oil containing $\textrm{F}^{18}$ with radioactivity of about 1 mC. The sampling time last for 60*s*, and we took the sampling data at equal time intervals for image reconstruction to obtain the fluid images in a more stable state. We denoised the images and the results are shown in Fig. 8.

In practical application, the fluid state (including whether there are cracks, irregular sections, etc.) can be observed through images to judge the internal conditions of the pipeline. In principle, the better the image quality, the higher the accuracy of the detection results.

From the different image effects under each model in the figure, we can see that the method proposed in this paper has a good denoising effect, and the image quality has been significantly improved. This method uses the extracted image internal information feature fusion generative adversarial network to denoise the image. Theoretically, the more complex the internal structure of the industrial part cavity and the more complex the flow field image, the better the denoising effect of this model.

## Conclusions

The main goal of this paper is to denoise the reconstructed industrial positron flow field image, aiming at small sample data in special fields. It can be seen from the results of simulation experiments and field experiments that the proposed model has a good denoising effect, especially in the actual application scenario, the denoising process preserves the details of the image, and achieves the denoising task well.

The image resolution currently used is $128 \times 128$.In the experiment, we tried to improve the pixels of the image during the reconstruction process and denoise the image with higher pixels. The model can also achieve the denoising effect, but the result is not satisfactory. The main reason is that there is too little sampling data, which leads to less pixel information in the reconstructed image with higher resolution. Information loss and image distortion will occur after de-noising. Therefore, how to obtain a higher resolution image is also a future research direction.

In conclusion, we have proposed a generative adversarial network for positron flow field images denoising based on “zero-shot” learning, Which is dedicated to solving the problem of poor image quality under the scarce samples in industrial positron detection, and making the denoised images more readable. The experimental results also prove the feasibility of the proposed method. In the future, we plan to segment the image, try to directly process the region of interest (ROI), or fuse different neural networks to directly process the reconstructed original data, so as to further improve the image quality of industrial positron flow field.

## Data Availability

The data used in the study comes from two parts: the simulation data comes from GATE and it is a special simulation software for PET/SPECT equipment based on Mento Carlo; the real data comes from cooperative enterprises. The datasets used and/or analysed during the current study available from the corresponding author on reasonable request.
